# Unveiling genetic basis of seedling emergence from deep soil depth under dry direct- seeded conditions in rice (*Oryza sativa* L.)

**DOI:** 10.3389/fpls.2024.1512234

**Published:** 2025-01-29

**Authors:** Vagish Mishra, Shilpi Dixit, Swati Tyagi, Challa Venkateswarlu, Pronob J. Paul, Anoop Kishor Singh Gurjar, Shalabh Dixit, Nitika Sandhu, Smita Kurup, Arvind Kumar, Pallavi Sinha, Vikas Kumar Singh, Uma Maheshwar Singh

**Affiliations:** ^1^ International Rice Research Institute, Los Baños, Philippines; ^2^ IRRI South Asia Regional Center, Varanasi, Uttar Pradesh, India; ^3^ IRRI South Asia Hub, Patancheru, Hyderabad, India; ^4^ School of Agricultural Biotechnology, Punjab Agricultural University, Ludhiana, India; ^5^ Plant Sciences and the Bioeconomy, Rothamsted Research, Harpenden, Hertfordshire, United Kingdom

**Keywords:** direct-seeded rice (DSR), quantitative trait loci (QTLs), deep sowing depth, seed germination, mesocotyl length, coleoptile length

## Abstract

Water scarcity and labor shortage pose significant challenges in rice farming. Direct-seeded rice (DSR) is an efficient method that conserves water, reduces labor costs, and allows for full mechanization of cultivation. However, variable planting depth in undulated field leading to deep/shallow sowing of rice seeds during mechanical sowing presents a major hurdle, as existing varieties lack tolerance to deep sowing. To address this, a mapping population comprising 150 F_4_ lines, derived from MTU 1010 and AUS295, was developed and phenotyped for emergence from deep soil depth-related traits, including days of emergence (DE), percent germination (PG), mesocotyl length (ML), and coleoptile length (CL). The correlation revealed that DE has a significant negative correlation with PG, ML, and CL, whereas PG, ML, and CL are all positively correlated with each other. The mapping population was genotyped with mid-density SNP assay (1k-RiCA), and a linkage map was established with 414 polymorphic SNP markers. A total of 16 QTLs were identified for four traits, with phenotypic variance explained (PVE) ranging from 6.63% to 19.6% in the WS22. These included 5 QTLs for DE, 3 QTLs for PG, 4 QTLs for ML, and 4 QTLs for CL. Out of 16 QTLs identified, 12 were major effect QTLs (*qDE_1.2_*, *qDE_1.3_*, *qDE_1.4_*, *qDE_2.1_*, *qDE_12_*, *qPG_2.1_, qPG_2.2_, qML_2.1_, qML_2.2_*, *qCL_1_*, *qCL_2.2_, qCL_2.3_*) and 4 were minor effect QTLs (*qPG_1_, qML_1.2_, qCL_2.1_*). During DS23 season, QTL analysis for DE and PG traits identified seven and three QTLs, respectively. Out of the ten QTLs identified in DS23 season, eight were stable across the season. This study reported 11 novel QTLs, while 7 had been previously reported. The study pinpointed three QTL hotspot regions: one on chromosome 1 (*qPG_1_*, *qCL_1_*) and two on chromosome 2 (*qPG_2.1_, qML_2.2_, qCL_2.1_*) and (*qPG_2.2_, qCL_2.2_*). Candidate gene analysis in the identified QTL regions found two genes associated with hormonal pathways: *OsSLR1* for gibberellin signaling and *OsSAUR11* for abscisic acid signaling. Additionally, one gene (*OsMT3a*) associated with early seedling vigor and another (*OsABA8ox1*) regulates germination through coleoptile growth. The identified QTLs, genes, and breeding lines from this study provide valuable resources for developing rice varieties with enhanced tolerance to deep soil emergence, making them well-suited for mechanized DSR systems.

## 1 Introduction

Direct-seeded rice (DSR) is a highly effective method for conserving water and labor resources in rice cultivation by eliminating the need for traditional puddling and transplanting (Balasubramanian and Hill, 2002). In DSR, rice seeds are sown directly into non-flooded soil, typically in a well-prepared seedbed, which allows rice plants to grow in aerobic conditions (Kumar and Ladha, 2011). DSR significantly decreases the water requirements for rice cultivation without continuous flooding and reduces labor requirements because of complete mechanical operation options. DSR is a valuable approach to practice in regions facing water and labor issues and offers potential economic and environmental benefits (Liu et al., 2015). However, despite the several advantages of DSR over TPR (transplanted rice), it also has some issues, such as non-uniform emergence, weed infestation, poor seedling establishment, being prone to lodge, etc (Lee et al., 2017; Singh et al., 2017; Baltazar et al., 2019; Yang et al., 2022). Dry-DSR is typically practiced in dry fields, where uniform seedling emergence is crucial for establishing uniform crop stands. Seed sown under dry DSR at shallow levels may lead to seedling damage by drought, birds, rodents, and a higher chance of crop lodging. Deep sowing has the advantage of addressing these issues and improving nutrient and water absorption from the deeper soil. However, it also presents a significant challenge for crop establishment due to poor germination, which is a limitation for the adoption of this technique. To overcome this issue, it is crucial to understand the genetic basic of the trait and develop rice varieties suitable for emergence from deep soil depth.

Previous studies have demonstrated that rice coleoptiles and mesocotyl are primarily responsible for seedling emergence (Zhan et al., 2020). They elongate during germination and emerge out above the soil surface under deep sowing conditions (Dilday et al., 1990). In soil-sand culture, growth of mesocotyl is greater than coleoptile, whereas its contrast is true under submergence (Wang et al., 2021). This exhibits the importance of mesocotyl length in early and uniform seedling emergence from deep soil depth (Alibu et al., 2012). Genotypes characterized by longer mesocotyl tend to exhibit quicker and more uniform emergence (Zhang et al., 2005; Zhan et al., 2020). Consequently, mesocotyl elongation stands out as a crucial trait for deep-emergence, a pivotal factor in the context of dry DSR cultivation (Wu et al., 2015; Lu et al., 2016; Zhao et al., 2018). Besides genetic factors, mesocotyl elongation can be influenced by environmental and physical factors, such as soil type, soil depth, moisture content, temperature, and light. At a certain extent, deep-sowing of rice seedlings leads to an increase in mesocotyl length (Wang et al., 2021). However, mesocotyl growth may be suppressed under the exposure of light (Osterlund et al., 2000; Feng et al., 2017; Lee et al., 2017). Light exposure leads to the upregulation of polyamine oxidase (*OsPAO5*), which inhibits mesocotyl growth and seedling emergence from soil surface (Lv et al., 2021). Rice mesocotyl length is induced by alteration of phytohormones including abscisic acid (ABA), brassinosteroids (BRs), ethylene (ETH), gibberellin (GA), and indole-3-acetic acid (IAA), but inhibited by jasmonate (JA), karrikin, and strigolactones (SLs) (Watanabe et al., 2001; Cao, 2005; Xiong et al., 2017; Sun et al., 2018; Zhan et al., 2020; Zheng et al., 2020).

To understand the genetic basis of seedling emergence from various soil depths, previous studies have conducted genome-wide association studies (GWAS) in various rice accessions, identifying thirteen QTLs and two major effect genes (Zhao et al., 2018). QTLs for coleoptile and mesocotyl elongation at standard soil depths. For example, Lee et al. (2017) identified three QTLs related to mesocotyl elongation under deep seeding condition at 5-cm soil depth Marker-trait associations (MTAs) for seedling emergence from deep sowing have also been identified through genome-wide association studies (GWAS) (Zhao et al., 2018; Menard et al., 2021; Sakhale et al., 2023). Specific QTLs, such as *qEML_1_*, *qEML_7_*, and genes like *OsGSR1* and *OsMTD1*, have been reported for these traits. However, there is limited research on seedling emergence from 8-cm deep sowing under dry DSR conditions. Identifying QTLs associated with deep soil emergence in dry DSR is essential for improving seedling establishment in mechanized DSR systems.

The present study aims to identify and characterize QTLs associated with emergence from an 8- cm soil depth under dry DSR conditions. The approach includes developing a mapping population from a cross between MTU 1010 and AUS295, phenotyping for deep sowing tolerance traits, and genotyping with a medium-density SNP panel (1k-RiCA). Additionally, candidate gene analysis will be conducted to understand the regulatory pathways underlying these traits. A list of promising entries, characterized by a high germination rate from deep sowing, along with associated markers, will also be provided for use by breeders in breeding programs.

## 2 Results

### 2.1 Phenotypic variation in RIL population for deep emergence trait

**Figure 1:**
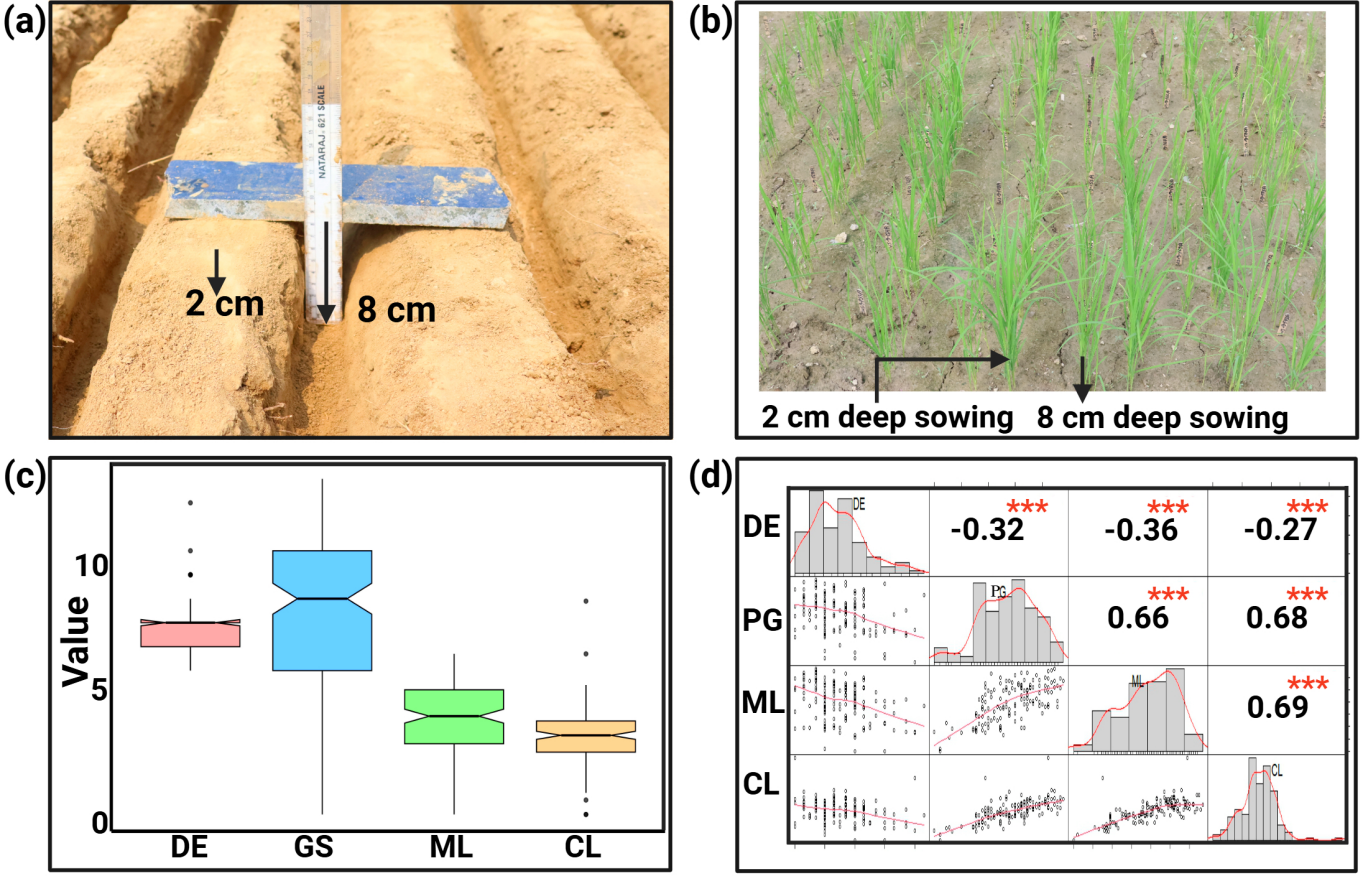
Seed sowing conditions, seedling emergence, phenotypic variation and trait correlation: **(A)** Seeds were sown at 8 cm seeding depth and at the depth of 2 cm for control sample. **(B)** Arrows highlight the emergence of seedlings from varying depths, 2 cm and 8 cm, across different entries. **(C)** Box plots in the same figure visually depict the phenotypic variation for various deep soil emergence-related traits: DE (days of emergence), Percent germination represented as number of germinated seedlings (GS), ML (mesocotyl length), and CL (coleoptile length) measures in cm. **(D)** Distribution curve sowing the range of variables, with pearson's correlations illustrating the relationships among, PG, ML, and CL. ^***^ indicating significant difference with a *p-value <*0.001.

Significant differences were observed among the parents and the RIL population for deep emergence traits (**Figures 1A–C**). Descriptive statistics analysis of phenotypic data from the mapping population for DE, PG, ML and CL during WS2022 indicates that the average value of DE varies from 6 to 13 days in the population, with MTU 1010 and AUS295 emerging at 13 and 8 days, respectively (**Table 1**; **Supplementary Table S1**). Both parents' seeds germinated from 8 cm depth, with AUS295 showing 89% germination and MTU 1010 displaying only 40% germination. Similarly, analysis from the DS2023 experiment for DE and PG, indicates that DE varies from 5 to 14 days in the population (**Table 1**; **Supplementary Table S1**). In the mapping population, PG ranged from 4.4% to 95.6% during WS2024 and 5 to 100 during DS2023. The range of ML in the population varies from 0.2 to 6.7 cm, with MTU 1010 and AUS295 having lengths of 2.4 and 6.1 cm, respectively. Similarly, the CL of the population ranges from 0.6 cm to 8.9 cm, while MTU 1010 and AUS295 had lengths of 1.8 and 3.9 cm, respectively. Overall, the results indicated a heritability of 98% for the traits studied. The frequency distribution for the traits showed varied patterns: DE displayed a left-skewed distribution, ML a right-skewed distribution, and both PG and CL followed a normal distribution (**Figure 1D**).

**Table 1:** Descriptive statistics of traits related to seedling emergence from deep soil depth.

^a^Traits	Season	Mean	^b^SD	^C^SE	Min.	Max.	Variance	Heritability
DE	WS22	7.59	1.05	0.10	6	13	1.11	98
PG	WS22	58.90	19.84	1.73	4.4	95.6	393.70	
ML	WS22	4.08	1.45	0.12	0.2	6.7	2.10	
CL	WS22	3.24	1.14	0.10	0.6	8.9	1.29	
DE	DS23	8.5	1.34	0.11	5	14	1.8	
PG	DS23	60.96	22.26	1.82	5	100	495.63	

Seedling emergence from (8 cm) soil depth related. WS, wet season; DS, dry season; ^a^traits DE (No.), PG (%), ML (cm) and CL (cm), ^b^standard deviation, ^c^standard error.

### 2.2 Correlation analysis among the traits

The correlation analysis of phenotypic data collected during WS2023 revealed a significant negative correlation between the DE and PG (r = - 0.32, p < 0.001) (**Figure 1D**). Similarly, DE also showed a negative correlation with ML (r = - 0.36, p < 0.001) and CL (r = -0.27, p < 0.001), suggesting that higher DE values corresponded to lower ML and CL values. In contrast, PG exhibited a strong and significant positive correlation with both ML (r = 0.66, p < 0.001) and CL (r = 0.68, p < 0.001), indicating that as PG increased, ML and CL also tended to increase. A similar trend was observed between ML and CL, with a highly significant positive correlation (r = 0.69, p < 0.001), reflecting the close association between these parameters at this depth. These correlations highlight the intricate relationships between DE, PG, ML, and CL at an 8 cm depth, suggesting that changes in one parameter are closely linked to variations in the others.

### 2.3 QTL analysis for deep emergence traits

To identify the genomic regions associated with traits for deep emergence, four component traits were selected for the study: DE, PG, ML, and CL. Among these, DE and PG are direct traits visible in the field, while ML and CL are indirect component traits that contribute to seedling emergence under deep soil conditions. For dry- DSR under deep seeding conditions, PG is considered a critical trait, as it directly impacts crop establishment. The positive correlation of PG with both ML and CL highlights their role in seedling emergence: ML helps push the seedling above the soil surface, while CL protects the growing shoot.

**Figure 2:**
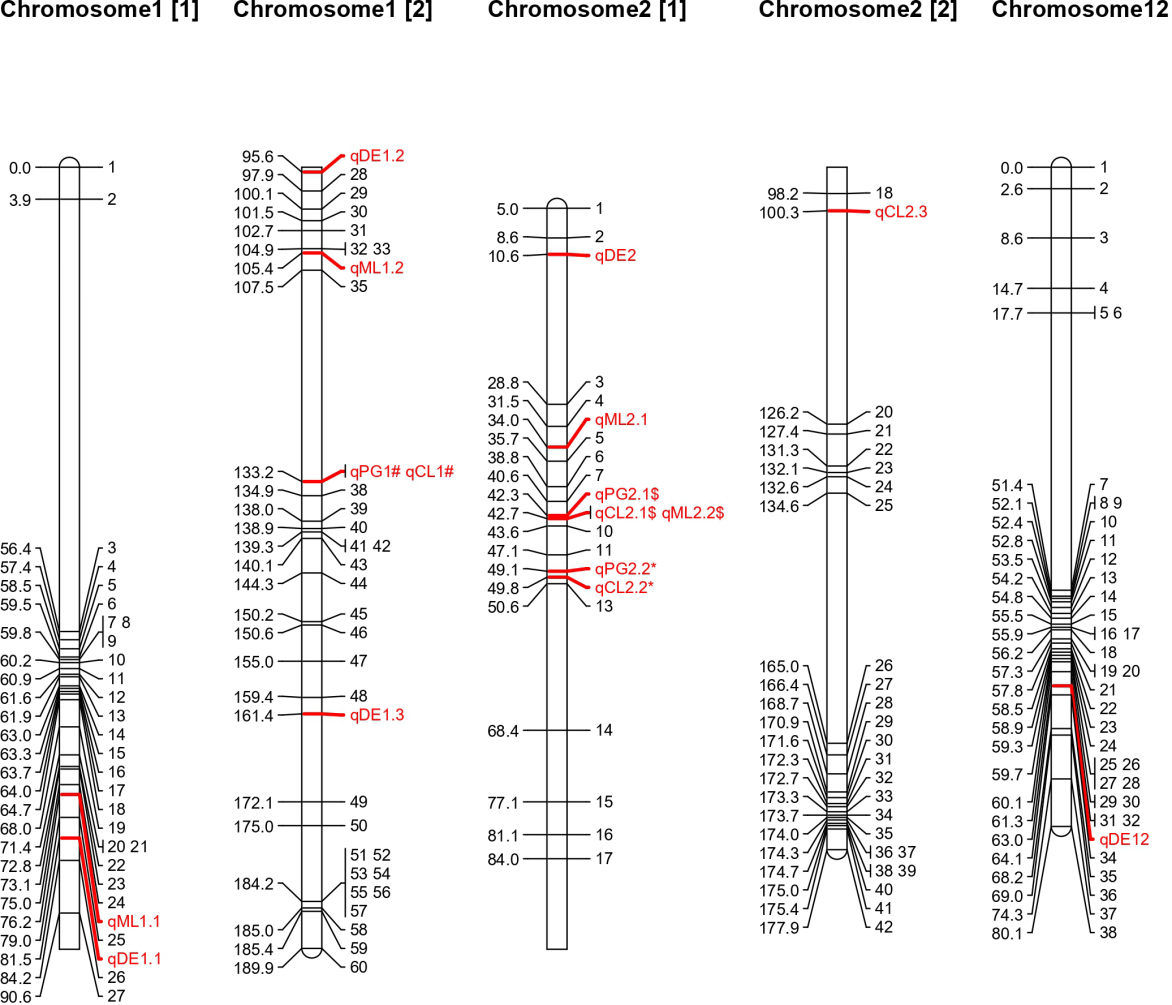
Chromosomal location of QTL detected for deep soil emergence related traits.

To identify genomic regions for these traits, genotyping was carried out using a mid-density SNP panel (1k-RiCA), which identified 414 polymorphic markers out of 1,094, with a polymorphism rate of 37.84% (**Supplementary Table S2**). A linkage map was constructed with these 414 markers across 12 linkage groups, spanning 1219.36 cM, with an average inter-marker distance of 2.94 cM. Sixteen QTLs associated with the four deep emergence traits from WS22 experiment were identified on chromosomes 1, 2, and 12 (**Table 2**). Of these, 5 QTLs are associated with DE, 3 with PG, 4 each with ML and CL. The phenotypic variance explained (PVE) value of these QTLs varies from 6.63 – 19.6%. QTL analysis was also conducted for DE and PG from the data generated during DS23 and identified seven and three QTLs respectively (**Supplementary Table S3** **;** **Supplementary Figure S1**). The PVE value of these QTLs varies from 9 – 19.6 %. QTLs with a strong effect are depicted in **Figure 2**. Interestingly, the favorable alleles contributing to these QTLs were derived from both parents. Specifically, 22 QTLs were contributed by AUS295, while four were contributed by MTU 1010. This finding aligns with the observed phenotypic differences between the two contrasting parents. The details of each trait are provided below:

**Table 2:** Quantitative trait loci identified for traits related to seedling emergence from deep soil depth.

S. No	Trait	Chr.	QTL	Marker interval/ position (bp)	Peak position (cM)	LOD	PVE (%) or R^2^	ADD
	QTLs identified during WS22							
1	DE	1	*qDE_1.2_*	5510350-6543217	81.5	2.8	12.5	-2.04
2	DE	1	*qDE_1.3_*	5064126-4307004	95.6	4.1	19.6	-3.38
3	DE	1	*qDE_1.4_*	26187514-23091103	161.4	4.3	19.6	-3.38
4	DE	2	*qDE_2.1_*	34072964-30345735	10.6	4.7	12.3	-1.36
5	DE	12	*qDE_12_*	7445812-7114076	63.0	3.3	12.5	-6.50
6	PG	1	*qPG_1_^#^*	36150523-35794891	133.21	3.9	9.05	0.47
7	PG	2	*qPG_2.1_^$^*	25949636-24887320	42.31	4.6	11.20	-2.04
8	PG	2	qPG_2.2_^*^	23852677-23396915	49.11	3.4	11.48	-3.38
9	ML	1	*qML_1.1_*	5408523-5510350	76.2	2.6	11.44	-3.38
10	ML	1	*qML_1.2_*	1532281-932866	105.4	2.7	6.27	-3.38
11	ML	2	*qML_2.1_*	30530432-28049479	34.1	*5.9*	15.33	-1.36
12	ML	2	*qML_2.2_^$^*	25949636-24887320	42.8	*5.2*	12.69	-6.50
13	CL	1	*qCL_1_^#^*	36150523-35794891	133.21	6.4	14.55	8.39
14	CL	2	*qCL_2.1_^$^*	25949636-23852677	42.71	3.6	6.63	0.53
15	CL	2	qCL_2.2_^*^	23852677-23396915	49.81	2.9	10.06	-0.49
16	CL	2	qC_L2.3_	7238793-6186335	100.2	3.7	9.05	0.64
	QTLs identified during DS23							
1	DE	1	*qDE_1.1_*	34508997-2726468	63.0	2.6	11.3	-1.5
2	DE	1	*qDE_1.2_*	5510350-6543217	81.5	2.8	12.5	-2.04
3	DE	1	*qDE_1.3_*	5064126-4307004	95.6	4.1	19.6	-3.38
4	DE	1	*qDE_1.4_*	26187514-23091103	161.4	4.3	19.6	-3.38
5	DE	2	*qDE_2.1_*	34072964-30345735	10.6	4.7	12.3	-1.36
6	DE	2	*qDE_2.2_*	103273-316859	164.1	3.1	13.1	-1.8
7	DE	12	*qDE_12_*	7445812-7114076	63.0	3.3	12.5	-6.50
8	PG	1	*qPG_1_*	36150523-35794891	133.21	3.9	9.05	0.47
9	PG	2	*qPG_2.1_*	25949636-24887320	42.31	4.6	11.20	-2.04
10	PG	2	*qPG_2.2_*	23852677-23396915	49.11	3.4	11.48	-3.38

DE, is Days of Emergence; PG, percent germination; ML, mesocotyl length; CL, coleoptile length; Chr, Chromosome; LOD, logarithm of odds, PVE, phenotypic variance explained; ADD, additive effect; ^#^common locus for PG/CL; ^$^common locus for PG/ML/CL; *common locus for PG/CL.

#### 2.3.1 QTLs for days of emergence

A total of 5 QTLs associated with DE were identified on chromosomes 1, 2, and 12, with LOD scores ranging from 2.8 to 4.7 during WS22 (**Table 2**). These QTLs exhibited major effects, with PVE values ranging from 12.3 % to 19.6%. The QTL with the lowest PVE (12.3%) was *qDE_2.1_* on chromosome 2, while the highest PVE (19.6%) was observed for *qDE_1.3_* and *qDE_1.4_* on chromosome 1. Chromosome 1 harbored the highest number (3) of QTLs for DE, followed by one QTLs on chromosome 2 and 12 respectively. Notably, all 5 QTLs were contributed by AUS295. During DS23, a total of seven QTLs associated with DE were identified across three chromosomes (1, 2, and 12) with LOD score ranging from 2.6 to 4.7. The QTL with lowest PVE value was 11.3 (*qDE_1.1_*) and highest PVE was 19.6 (*qDE_1.3_* and *qDE_1.4_*). Chromosome 1 harbor maximum number of QTLs and all the seven QTLs are contributed by AUS294.

#### 2.3.2 QTLs for percentage germination

Three QTLs were identified for PG on chromosomes 1 and 2, with LOD values ranging from 3.4 to 4.6 during WS22. The QTL on chromosome 1, *qPG_1_*, exhibited a PVE of 9.05%, while *qPG_2.1_* and *qPG_2.2_* on chromosome 2 showed PVEs of 11.2% and 11.48%, respectively. The major QTL, *qPG_2.1_* and *qPG_2.2,_* was contributed by AUS295, while *qPG_1_* MTU 1010 contributed (**Table 2**). During DS23, same 3 QTLs associated with PG were identified, with LOD value ranging from 3.4 to 4.6 and PVE ranging from 9.05 (*qPG_1_*) to 11.48 (*qPG_2.2_*) (**Table 2**).

#### 2.3.3 QTL for mesocotyl length from deep sowing condition

A total of four QTLs were identified for ML on chromosomes 1 and 2, with an LOD range of 2.6 to 5.9 (**Table 2**). Two QTLs, on chromosome 1 (*qML_1.1_* and *qML_1.2_*) showed PVEs of 11.44 and 6.27%, respectively while on chromosome 2 (*qML_2.1_* and *qML_2.2_*) showed PVEs of 15.33 and 12.69%, respectively. All 5 QTLs were contributed by AUS295.

#### 2.3.4 QTL for coleoptile length from deep sowing condition

A total of four QTLs were identified for CL on chromosomes 1 and 2, with LOD ranging from 2.9 to 6.4 (**Table 2**). The *qCL_1_* was identified on chromosome 1 and had a PVE of 14.55%. On the other hand, *qCL_2.1_*, *qCL_2.2_*, and *qCL_2.3_* located on chromosome 2 had a PVE of 6.63, 10.06, and 9.05%, respectively. Interestingly, *qCL_2.2_* was contributed by AUS295, but the rest of the three QTLs, *qCL_1_*, *qCL_2.1,_* and *qCL_2.3_*, were inherited from the MTU 1010.

### 2.4 Candidate gene analysis from the identified QTL regions

The QTLs identified for deep emergence-related traits, including DE, PG, ML and CL were further analyzed to identify the presence of known genes contributing to the ability of rice seedlings to emerge from deep soil. A total of 13 genomic/QTL regions were analyzed for finding target related genes (**Supplementary Table S3**). Through further analysis and literature mining, three putative genes-*OsSLR1*, *OsSAUR11*, and *OsMT3a*-were identified (**Table 3**). The *OsSLR1* (slender rice1) was found within the *qDE_1.1_* region, which plays a significant role in gibberellin signaling, a crucial hormone involved in seed germination and early seedling growth. Similarly, *OsSAUR11*, located in the QTL region associated with *qPG_2.1_*, is involved in auxin signaling, another key phytohormone regulating plant growth and development. The expression of *OsSAUR11* supports robust early seedling growth, which is critical for deep soil emergence. Interestingly, *OsMT3a*, a metallothionein gene, was identified within the QTL regions associated with *qML_1.1_*, *qML_2.2_* and *qCL_2.1_*. This gene is known for its role in abiotic stress tolerance, such as oxidative stress and reported enhances seedling vigor, particularly under challenging conditions like deep sowing, where seedlings need to overcome additional environmental stressors to emerge. These genes are integral to regulating early seedling emergence, particularly under conditions where seeds are sown deeper into the soil. The identification of these genes within QTL regions for deep emergence-related traits highlights their potential roles in controlling key processes such as phytohormone synthesis and stress response. By regulating hormones like gibberellin and auxin, as well as contributing to stress tolerance, these genes are likely to be crucial in improving rice varieties for DSR systems, where deep sowing can otherwise pose significant challenges to seedling establishment.

**Table 3:** Candidate genes identified from mapped QTL regions.

S. No.	QTLs*	Locus ID	Gene name	Position	Description
1	*qDE_1.1_*	Os01g0646300	*OsSLR1*	chr01:26044168.26045843	Key gene involved in the gibberellin (GA) signaling pathway in rice, which is essential for seed germination (Mo et al., 2020; Kim et al., 2023).
2	*qPG_2.1_* *qML_2.2_* *qCL_2.1_*	Os02g0643800	*OsSAUR11*	chr02:25878905.25879875	Auxin-responsive gene, plays a crucial role in root development (Xu et al., 2023)
3	*qML_1.1_*	Os01g0200700	*OsMT3a*	chr02:5408523.5510350	Plays a significant role in enhancing seed germination, particularly under stress conditions (Mekawy et al., 2020)
4	*qML_2.1_*	Os02g0703600	*OsABA8ox1*	chr02:28995717.28998452	Play role in seedling germination through regulating coleoptile growth (Mao et al., 2017)

*QTLs identified in current study; DE, is days of emergence; PG, percent germination; ML, mesocotyl length; CL, coleoptile length.

## 3 Discussion

The present study provides critical insights into the genetic mechanisms governing seedling emergence from deep soil depths, particularly in dry- DSR. By evaluating a RIL population derived from AUS295 and MTU 1010, significant phenotypic differences were observed between the parents, particularly for traits related to deep emergence, such as DE, PG, ML and CL (**Supplementary Table S1**). These variations reflect the genetic diversity present in the mapping population, which was further explored through the identification of QTLs associated with these traits (**Figure 2**; **Table 2**). This study not only confirms the role of mesocotyl and coleoptile elongation in improving seedling emergence under deep-sowing conditions but also advances our understanding by pinpointing specific genomic regions and candidate genes contributing to these traits.

The study revealed significant phenotypic variations in deep emergence traits. DE in AUS295 was observed eight days after sowing; however, MTU 1010 took thirteen days to emerge after sowing. Significant variation in the DE indicated that there was substantial genetic variation between both parents and donor parent. AUS295 exhibited early emergence. AUS295 demonstrated superior germination/PG from an 8 cm depth, achieving 89%, compared to MTU 1010 with 40% germination rate whereas in the mapping population PG varies from 4.4-95.6% (**Supplementary Table S1**). These findings are consistent with previous studies (Wu et al., 2015; Lu et al., 2016; Lee et al., 2017; Zhao et al., 2018; Zhang et al., 2023; Sakhale et al., 2023) that reported enhanced mesocotyl length as a contributing factor to early seedling emergence under deep-sowing conditions.

The present study reveals significant variability in ML and CL within the population, ranging from 0.2 to 6.7 cm and 0.6 to 8.9 cm, respectively. Notably, MTU 1010 and AUS295 exhibited distinct lengths, with MLs of 2.4 cm and 6.1 cm, and CLs of 1.8 cm and 3.9 cm, respectively. The high heritability estimate of 98% underscores the genetic control over these traits. The frequency distribution analysis indicated diverse patterns: DE showed a left-skewed distribution, ML a right skewed distribution, while PG and CL followed a normal distribution (**Figure 1D**). The observed negative correlations between DE and PG, DE and ML, and DE and CL, alongside positive correlations between PG and ML, PG and CL, and ML and CL, highlight the complex interplay among these traits. These findings suggest that selective breeding for one trait could have cascading effects on others, either beneficial or detrimental.

Our findings corroborate previous research, affirming the direct relationship between mesocotyl and coleoptile lengths and seedling emergence under deep-sowing conditions. Enhanced elongation of these structures is consistently linked to improved seedling emergence and establishment, as documented by Turner et al. (1982), Murai et al. (1995), Luo et al. (2007), Chung (2010), and Alibu et al. (2012). Specifically, Murai et al. (1995) demonstrated this relationship in lines sown at soil depths up to 7 cm, while Chung (2010) observed similar positive correlations at a 5 cm seeding depth in Korean weedy rice accessions. Alibu et al. (2012) reported that upland rice accessions with longer mesocotyls exhibited superior emergence compared to those with longer coleoptiles under dry direct-seeding conditions. Furthermore, Lu et al. (2016) found that rice accessions with longer mesocotyls had higher emergence rates than those with shorter mesocotyls at sowing depths of 2 and 5 cm. These results underscore the importance of mesocotyl and coleoptile lengths in breeding programs aimed at improving seedling emergence and establishment, particularly under challenging deep-sowing condition.

Several studies attempted to map QTLs for ML and CL under standard sowing conditions, by utilizing phenotypic data generated from different phenotyping methods, such glass tube method (Katsuta-Seki et al., 1996), the slant-board test (Redona and Mackill, 1996), filter paper with distilled water (Huang et al., 2010), agar medium (Lee et al., 2012), and filter paper on agar medium (Xie et al., 2014), plastic pot (Lee et al., 2017), field experiment (Zhao et al., 2018), and petri dish (Zhang et al., 2023). However, only a few studies have investigated QTLs/MTAs under deep sowing conditions. In the current study, a total of 16 QTLs linked to deep emergence traits across various chromosomes were detected during WS22 season and 10 QTLs for DE and PG during DS23. These QTLs exhibited a range of PVE, from 6.27% to 19.6%. Notably, AUS295 contributed a higher number of favorable alleles (22 QTLs) compared to MTU 1010 (4 QTLs) in both season, which corresponds with the phenotypic distinctions observed between the parental lines. To deploy this finding into a breeding program, we present a table (**Supplementary Table S4**) listing entries with over 80% germination from deep soil depths, along with the presence or absence of QTLs. Given the availability of genotype, QTL, and marker data, this information can be directly applied in a marker-assisted breeding program to develop varieties that are tolerant to deep soil conditions, specifically for dry DSR cultivation.

Among the 16 identified QTLs associated with deep emergence traits, 5 were specifically linked to DE during WS22. Noteworthy are three QTLs, *qDE_1.2_*, *qDE_1.3_* and *qDE_1.4_*, located on chromosome 1, which were particularly associated with early seedling emergence (**Table 2**). These QTLs (*qDE_1.2_*) exhibited a PVE of 12.5% while (*qDE_1.3_* and *qDE_1.4_*) showed 19.6% PVE indicating their significant roles in determining the early emergence capability of seedlings from deep soil depths. Seven QTLs were identified during DS23, five of which had already been reported in WS22. Two new QTLs, *qDE_1.1_ and qDE_2.2_*, identified during DS23, were not considered for further analysis due to their expression in only one season. *OsSLR1* gene in QTL- *qDE_1.2_* identified in the current study (**Table 3**), has role in seedling germination. *OsSLR1* is a key gene involved in the gibberellin (GA) signaling pathway in rice, which is essential for seed germination (Mo et al., 2020; Kim et al., 2023). GA perception by the GA receptor *GID1* leads to the degradation of the *OsSLR1* protein, triggering GA-associated responses such as mesocotyl elongation, shoot elongation, seed germination (Mo et al., 2020). *OsSLR1*, the only DELLA protein in rice, acts as a repressor in GA signaling pathways. It regulates mesocotyl elongation by inhibiting the expression of genes that promote growth in the absence of GA. The interaction between *OsSLR1* and *OsPIL14*, a transcription factor that promotes cell elongation, is essential for controlling mesocotyl length.

Three QTLs (*qPG_1_*, *qPG_2.1_*, *qPG_2.2_*) identified for PG have a contribution of 9.05% to 11.48% of the PVE during WS22 and 9.05% to 11.48 % during DS23. Similar to the QTL position of *qPG_2.2_* identified in the current study, Edzesi et al. (2023) also reported a QTL *qMel-2*, which helps in mesocotyl elongation and thus promotes seed germination. The study suggests that during germination, mesocotyl elongates and pushes the shoot tip to emerge from the soil (Zhan et al., 2020). Kanno et al. (2023) noted that deep sowing increases the PG in drought/dry soil surfaces compared to seeds sown at a normal depth (1- 2 cm). The germination percentage under deep sowing is a crucial trait, particularly because in fields with low plant density resulting from deep sowing, the yield can be further diminished if weeds are not effectively managed, leading to increased weed presence and reduced crop coverage (Ahmed et al., 2014; Gealy and Duke, 2017). In the common QTL region (*qPG_2.1_*, *qML_2.2_*, *qCL_2.1_*), the auxin-responsive gene *OsSAUR11* is located (**Table 3**). This gene plays a pivotal role in root development, significantly contributing to the development of a robust root system in rice plants. According to Xu et al., 2023 *OsSAUR11* plays a crucial role for rice germination, enhancing root development and drought resistance. Overexpression of *OsSAUR11* in transgenic rice significantly increased the ratio of deep rooting, facilitating water absorption from deeper soil layers during drought conditions. This enhancement is vital for successful seed germination and seedling establishment under water-limited environments.

The four QTLs (*qML_1.1_*, *qML_1.2_*, *qML_2.1_*, *qML_2.2_*) identified for ML contribute 11.44, 6.27, 15.33 and 12.69% of PVE, respectively. Lee et al. (2017) reported an increase in ML and CL of various rice genotypes from deep sowing in BILs and CSSLs population developed for QTL study under deep soil depths (3-10 cm). Zhao et al. (2018) conducted a GWAS on 621 cultivated rice accessions from the 3000 Rice Genome Project (3K-RGP) and identified 13 QTLs for ML traits under dry DSR conditions. Similar to PG *OsSAUR11* (auxin-responsive gene) is located on the ML QTL (*qML_2.2_*) (**Table 3**) which plays a crucial role in root development, significantly contributing to the development of a root system in rice plants. Zhang et al. (2023) conducted a study using 144 RILs and 2,828 bin-markers to identify QTLs associated with mesocotyl length in a growth chamber experiment. Sixteen QTLs were identified across various chromosomes, with seven constant QTLs. Among these, the major QTL, *qML3a*, was re-identified using composite interval mapping. Detailed analysis of the *LOC_Os03g50550* gene revealed its role as a strong candidate for mesocotyl elongation, encoding a mitogen-activated protein kinase.

Four QTLs (*qCL_1_*, *qCL_2.1_*, *qCL_2.2_*, and *qCL_2.3_*) identified for CL explained 14.55, 6.63, 10.03, and 9.05% of the PVE, respectively. Out of the four QTLs identified for CL in our study, only one was contributed by the donor parent AUS295, while the rest were derived from the recipient parent. This could be attributed to factors such as epigenetic effects, differences in trait architecture, or complex genetic interactions. Similar to the current findings, Lee et al. (2017) also identified two QTLs for CL at 7 cm and 10 cm soil depths. CL is directly related to seedling emergence in deep seeding, and enhanced coleoptile elongation is associated with better seedling emergence and establishment (Turner et al., 1982; Murai et al., 1995; Luo et al., 2007; Chung, 2010; Alibu et al., 2012). Murai et al. (1995) reported that a relationship exists between seedling emergence ability, leaf, and CL of dwarf lines under 7 cm soil depth. Chung (2010) reported that an increase in CL promotes seedling emergence. Alibu et al. (2012) suggested the importance of long coleoptiles in seedling emergence under the dry direct-seeding condition. In addition to seeding depth, under dry seeding, the CL can be affected by moisture content. Under submergence, coleoptile growth was stimulated (Takahashi, 1978; Alibu et al., 2011). At QTL *qCL_2.1_* position *OsSAUR11* gene was found (**Table 3**), encoding a small auxin-up RNA (SAUR) protein, plays a crucial role in coleoptile elongation during seedling development in rice. The SAUR proteins, including *OsSAUR11*, are part of a large multigene family that responds rapidly to auxin application, a phytohormone known for its pleiotropic effects on plant growth and development (Xu et al., 2023). The identified QTLs provide breeders with valuable genetic information for improving root and shoot elongation, critical factors for seedling emergence from various sowing depths.

The co-localization of QTLs identified in the current study with those from previous reports revealed significant overlaps. Among the 16 identified QTLs, seven (*qDE_2.1_*, *qPG_1_*, *qPG_2.1_*,*qPG_2.2_*, *qML_1.2_*, *qCL_1_* and *qCL_2.1_*) were found to be located in the regions previously reported (**Table 4**). Specifically, the QTL position of *qDE_2.1_* on chromosome 2 has been previously reported for shoot dry weight (*qSDW_2_* and *qSDWT_2.1_*) (Sakhale et al., 2023). Similarly, QTLs for Shoot dry weight (*qSDW_30_*) and for crop growth regulation (*qCGR_30_*) were reported by Bharamappanavara et al. (2020). Notably, *qPG_2.2_* for percent germination on chromosome 2 was located in the same region as previously reported *qMel_-2-2_* reported for mesocotyl length (Edzesi et al., 2023). These findings underscore the significance of the identified QTL regions, as they are associated with multiple traits related to deep emergence characteristics.

**Table 4:** Overlapping of QTLs identified for deep soil depth emergence-related traits with previous studies.

S.No	Trait	Chr.	QTL	LOD	R2	ADD	QTL/genes	Reference
QTLs identified in the current study							QTLs previously reported	
1	PG	2	*qPG_1_*	3.9	9.05	0.47	*qML_1.4_* for ML; *qSDW_30_* and *qCGR_30_* for SDW and CGR	Wang et al., 2021; Bharamappanavara et al., 2020
2	CL	2	*qCL_1_*	6.4	14.55	8.39	*qMl_1.4_* for ML	Wang et al., 2021
3	PG	2	*qPG_2.2_*	3.4	11.48	-3.38	*qMel_2-2_* for ML	Edzesi et al., 2023
4	ML	1	*qML_1.2_*	2.7	6.27	-3.38	*qML_1.2_* for ML	Wang et al., 2021; Lu et al., 2016
5	PG	2	*qPG_2.1_*	4.6	11.2	-2.04	*qML_2C_*	Zhang et al., 2023
6	CL	2	*qCL_2.1_*	3.6	6.63	0.53	*qML_2C_*	Zhang et al., 2023
7	DE	2	*qDE_2.1_*	4.7	12.3	-1.36	*qSDW_-2.1_*	Sakhale et al., 2023

DE, is days of emergence; PG, percent germination; ML, mesocotyl length; CL, coleoptile length; SDW, shoot dry weight; crop growth rate, CGR; Chr, Chromosome; LOD, logarithm of odds, PVE, phenotypic variance explained; ADD, additive effect.

The analysis of 16 QTLs led to the identification of four promising candidate genes from five QTL regions (**Table 3**). Among these, two genes (*OsSLR1* and *OsSAUR11*) are involved in phytohormone pathways, specifically gibberellin and auxin. Another gene, *OsMT3a*, is associated with seed germination under multiple abiotic stress conditions, while *OsABA8ox1* is linked to seed germination through coleoptile growth. According to Ueguchi-Tanaka et al. (2008), the *OsSLR1* protein serves as a repressor in the gibberellin signaling pathway. When the plant GID1 receptor perceives GA, it leads to the degradation of *OsSLR1* through the action of the F-box protein GID2. This degradation releases the repression on GA signaling, allowing for processes such as seed germination. *OsSAUR11*, an auxin-responsive gene, plays a crucial role in root development (Xu et al., 2023). Although knocking out *OsSAUR11* does not significantly affect deep rooting, its overexpression significantly enhances the development of a robust root system in rice plants. This gene’s expression is induced by auxin and drought, and it is localized in both the plasma membrane and cell nucleus, enhancing the plant’s ability to cope with drought conditions, which is critical during germination and early growth stages. The *OsMT3a* gene, plays a significant role in enhancing seed germination, particularly under stress conditions (Mekawy et al., 2020). Overexpression of *OsMT3a* has been shown to improve the germination rate of transgenic plants under various abiotic stresses, such as salinity, drought, and heavy metal exposure. By enhancing the plant’s stress tolerance, *OsMT3a* contributes to more robust seed germination and seedling establishment, especially in challenging environmental conditions. The *OsABA8ox1* gene regulates seed germination in rice by modulating coleoptile growth through the catabolism of abscisic acid, thereby promoting the seedling germination via coleoptile growth (Mao et al., 2017). These findings highlight the significance of the identified QTL regions, as they are associated with multiple traits related to deep emergence characteristics. The integration of these QTLs/candidate genes into breeding programs could enhance the development of rice varieties with improved germination from deep sowing and greater resilience to environmental stresses.

## 4 Conclusion

This study reveals considerable genetic diversity in deep emergence traits (DE, PG, CL, ML) within parental lines and their RIL population. Our results underscore the importance of increased mesocotyl and coleoptile lengths in enhancing early seedling emergence under deep sowing. Correlation analysis highlights the interconnected nature of these traits, demonstrating complex relationships among percentage germination, deep emergence, coleoptile length, and mesocotyl length. AUS295 contributed a greater number of favorable alleles than MTU 1010, consistent with observed phenotypic differences. Of the 16 identified QTLs, 11 are novel, while the remaining are found in previously reported regions, offering new insights into the genetic architecture for deep sowing tolerance. Notably, certain QTL hotspots for *qPG_2.1_*, *qML_2.2_*, and *qCL_2.1_* exhibit pleiotropic effects. Additionally, four candidate genes linked to seedling emergence from deep soil, including those in phytohormone signaling and metallothionein pathways, illuminate the complex regulatory networks that govern seed germination and emergence from deep soil depths. These findings provide valuable genetic resources for breeders targeting enhanced seed emergence from deeper soil depths in dry DSR systems.

## 5 Materials and methods

The experiment was conducted at the International Rice Research Institute- South Asia Regional Center (ISARC), Varanasi, Uttar Pradesh, India, from the dry season 2020 (DS20) to the dry season 2023 (DS23) under experimental field conditions.

### 5.1 Planting material

The mapping population utilized in this study comprised 150 F_4:5_ RILs generated through the single seed descent (SSD) method. These lines originated from a cross between MTU 1010 (a high yielding *indica* rice variety) as the recipient and AUS295::IRGC 29083-1 (an upland *aus* genotype with long mesocotyl and deep emergence characteristics) as the donor. The crossing program commenced in the wet season 2019 (WS19) at the IRRI South Asia hub, ICRISAT campus, Patancheru, Telangana, with subsequent experimental procedures conducted at ISARC, Varanasi. The hybridity of the F_1_ plants was verified using a set of 10 quality marker sets (Intertek, Hyderabad), and positive F_1_ plants were advanced to the F_3_ generation through the SSD method.

### 5.2 Phenotyping of mapping population

Phenotyping for deep emergence traits was performed at F_4:5_ generations. Before the experiment, the seed germination of a random sample of 150 entries was examined in the seed germinator (Remi, India) at a temperature of 32°C and a relative humidity of 65%. Seed germination was recorded on the 5^th^ day of seed sowing in a petri dish. After ensuring good seed viability, ten seeds from each entry were manually sown in an 8-cm deep furrow and covered by soil after seeding. This experiment was performed in three replicates in field conditions across two seasons: the wet season of 2022 (WS22) and the dry season of 2023 (DS23), under dry DSR at ambient weather condition (**Supplementary Table S5**). During WS22, four traits- DE, PG, ML, and CL were recorded, whereas during DS23, data were collected only for two traits: DE and PG. Control experiments were grown parallel to the edge of the deep sowing furrow at a depth of 2 cm (**Figures 1A, B**). In the field experiment, we initiated daily counts of seedling emergence commencing on the fifth day post-sowing. Based on the time taken for seedlings to emerge, we estimated the total count of emerged seedlings. Additionally, we derived two important traits from this data: DE and PG. At 28 days after sowing, three emerged seedlings from each plot (if available) were excavated and cleaned under running tap water without disturbing the roots, followed by measurements of ML and CL (**Supplementary Table S1**).

### 5.3 Genotyping of mapping population

Dehusk seeds of F_3_ of RILs (150) and their parents were placed in a 96-deep well plate for 1k-RiCA genotyping (Arbelaez et al., 2019). Genotyping of samples was carried out using Diversity Arrays Technology (DArT) sequencing platform with 1094 SNP markers by Intertek, Hyderabad, India (https://www.intertek.com/agriculture/agritech/). In the parental polymorphism survey, out of 1094 markers, 414 were found to be polymorphic in the current study. These polymorphic markers were used for linkage map construction.

### 5.4 QTL analysis

#### 5.4.1 Linkage map construction

The linkage map was constructed using QTL ICI mapping software v 4.2 (Meng et al., 2015) (www.isbreeding.net). The grouping and ordering of markers were carried out using a regression mapping algorithm with a maximum recombination frequency of 0.4 at a minimum logarithm of odds (LOD) value of 2.5 using the commands “LOD groupings” and “create groups for mapping” into respective linkage groups (LG). The Kosambi map function was used for the construction of genetic maps and the calculation of map distance from recombination fractions. After developing the framework genetic maps with the marker orders, the unmapped markers were integrated into different linkage groups at recombination frequencies up to 50% using the ripple command. The resultant genetic maps were visualized using IciMapping version 4.2. The linkage map was 1219.36 cM in length (Kosambi, 1944), with a mean interval length of 2.94 cM (**Supplementary Figures S1**, **S2**).

#### 5.4.2 QTL mapping

QTL mapping was done with Windows QTL Cartographer version 2.5 (Silva et al., 2012). The composite interval mapping method (CIM) was performed with 1000 permutations and a significance level of 0.01, along with the standard model (model 6) of composite interval mapping with forward and backward regression methods. The QTLs with a threshold of >2.5 LOD were used as criteria for declaring the QTL. The graphics showing QTL location were obtained from Windows QTL cartographer v2.5. The standard procedure for QTL nomenclature is “The Committee on Gene Symbolization, Nomenclature, and Linkage” (CGSNL) of the Rice Genetic Cooperative (McCouch and CGSNL (Committee on Gene Symbolization, Nomenclature and Linkage, Rice Genetics Cooperative), 2008). Comparison of QTLs with previously reported QTLs was carried out using the Rice Annotation Project Database (RAP-DB), Gramene QTL database, and research publications.

### 5.5 Candidate gene analysis from the identified QTL regions

The QTLs for deep emergence traits, such as DE and CL were further analyzed to identify the presence of already reported genes associated with seedling emergence in rice under deep sowing conditions. Candidate genes within identified QTLs regions were retrieved from the RAP DB database (http://rapdb.dna.affrc.go.jp), and additionally, literature mining was also performed to identify genes previously reported to be linked with seedling emergence trait. Genes falling within the identified QTLs regions were shortlisted as potential candidates associated with the target traits (**Table 2**).

### 5.6 Statistical analysis

Deep soil depth emergence experiments were conducted in 3 replicates. Analysis of variance (ANOVA) was conducted using the lme4 package (Bates and Sarkar, 2007) in R software version 4.2.1. The correlation analysis was performed by the Pearson method and visualized using the “ggplot2” and “performance analytics” packages in R (**Figures 1C, D**).

## Data Availability

The original contributions presented in the study are included in the article/**Supplementary Material**. Further inquiries can be directed to the corresponding author.
