# Supplementary material for: Unveiling genetic basis of seedling emergence from deep soil depth under dry direct- seeded conditions in rice (Oryza sativa L.)
Source: Front Plant Sci. 2025 Jan 29;15:1512234. doi: 10.3389/fpls.2024.1512234 (PMC11814172; doi:10.3389/fpls.2024.1512234)
Supplement: Supplementary file 7 [file Image2.pdf]

QTL position using WS2022 phenotypic data

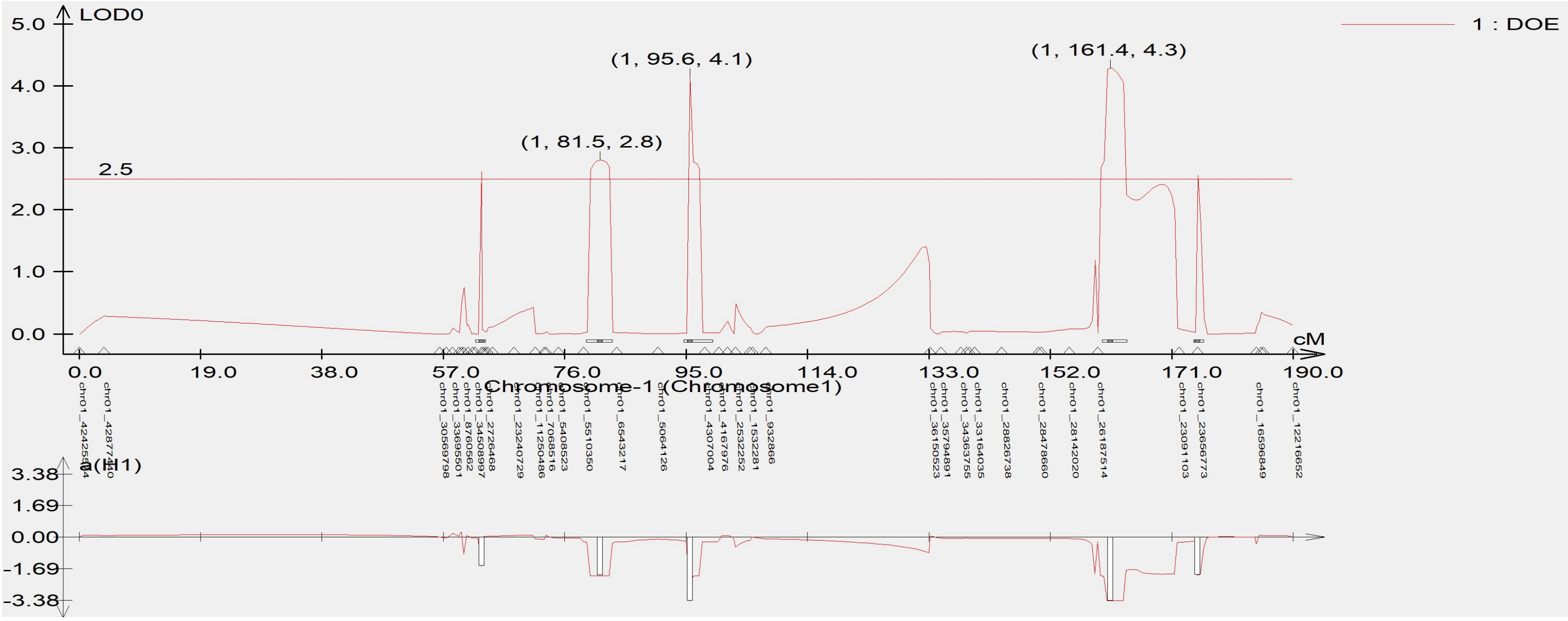

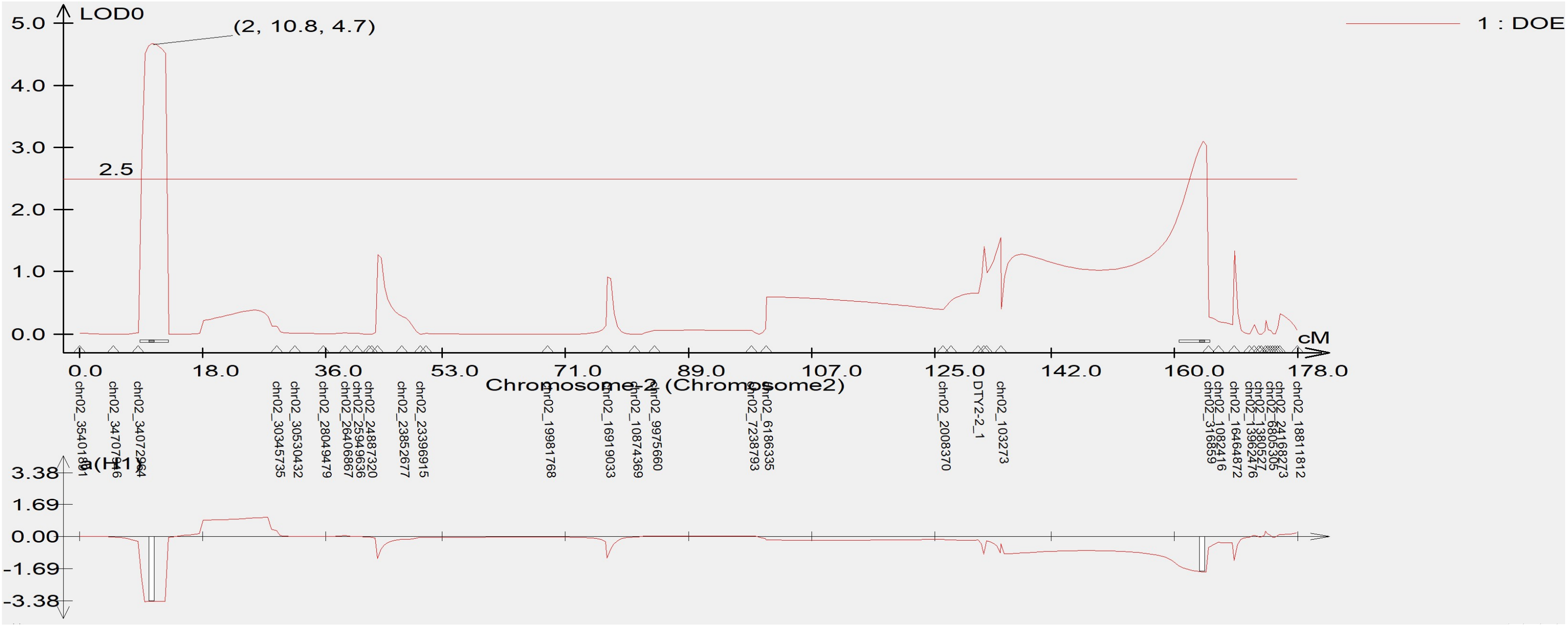

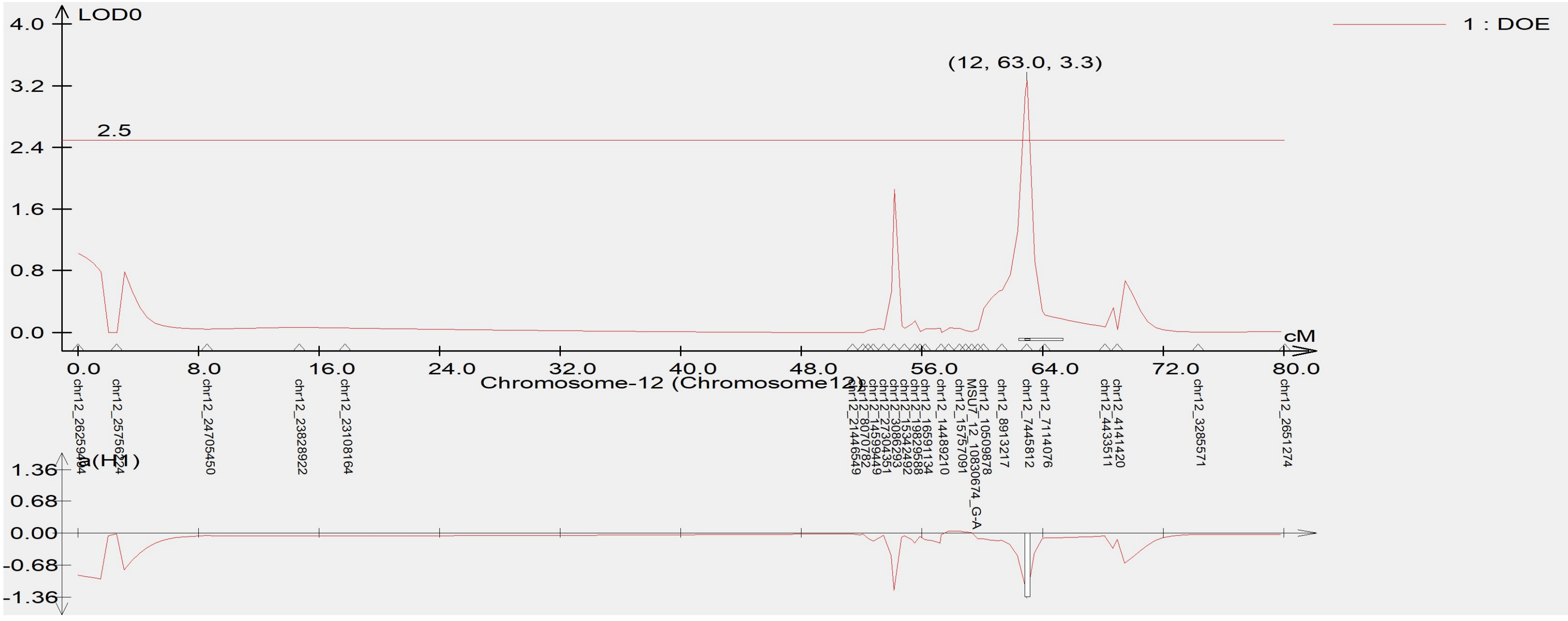

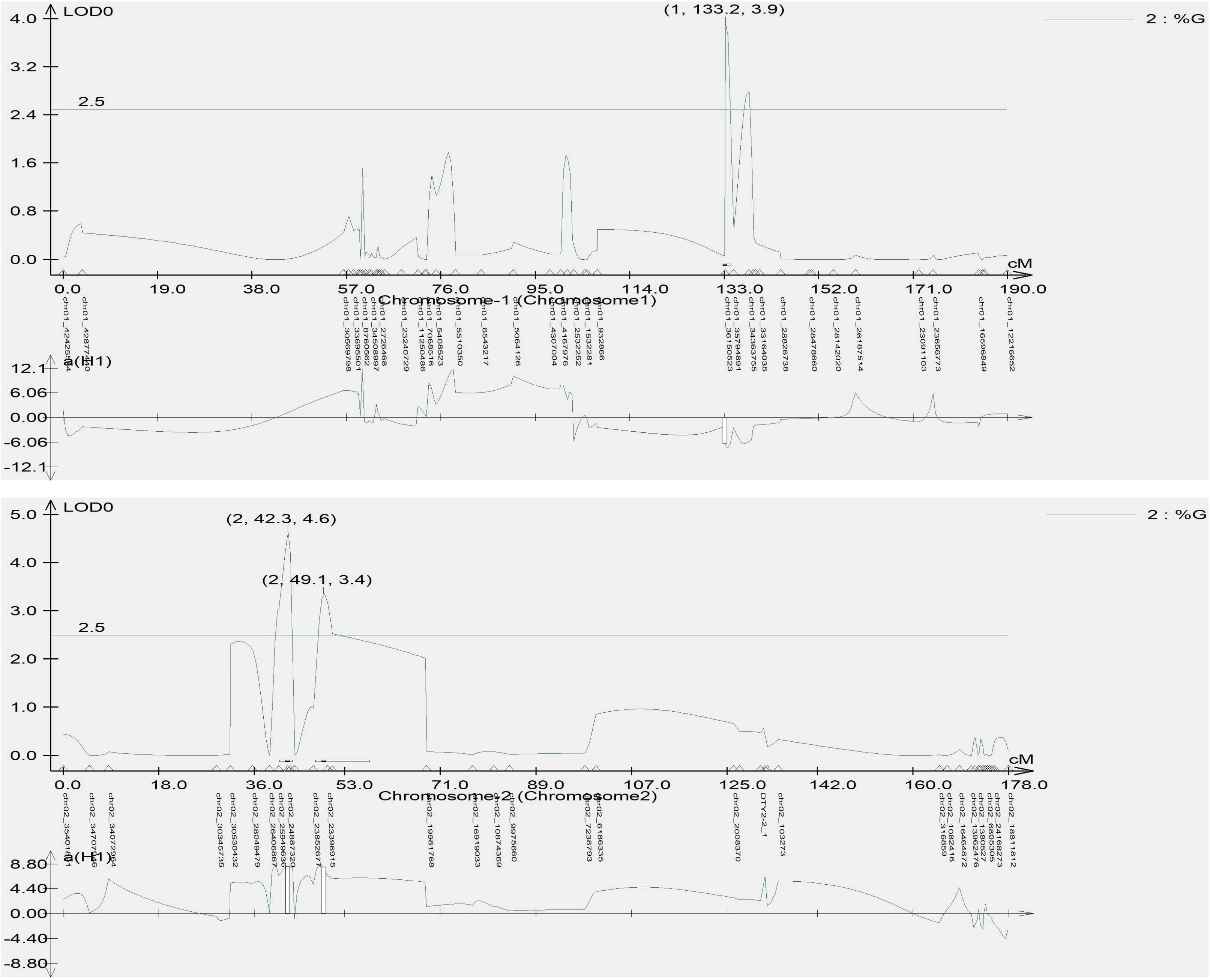
LOD0
(1, 133.2, 3.9)
2 : %G
2.5
Chromosome-1 (Chromosome1)
cM
a(H1)
LOD0
(2, 42.3, 4.6)
(2, 49.1, 3.4)
2 : %G
2.5
Chromosome-2 (Chromosome2)
cM
a(H1)

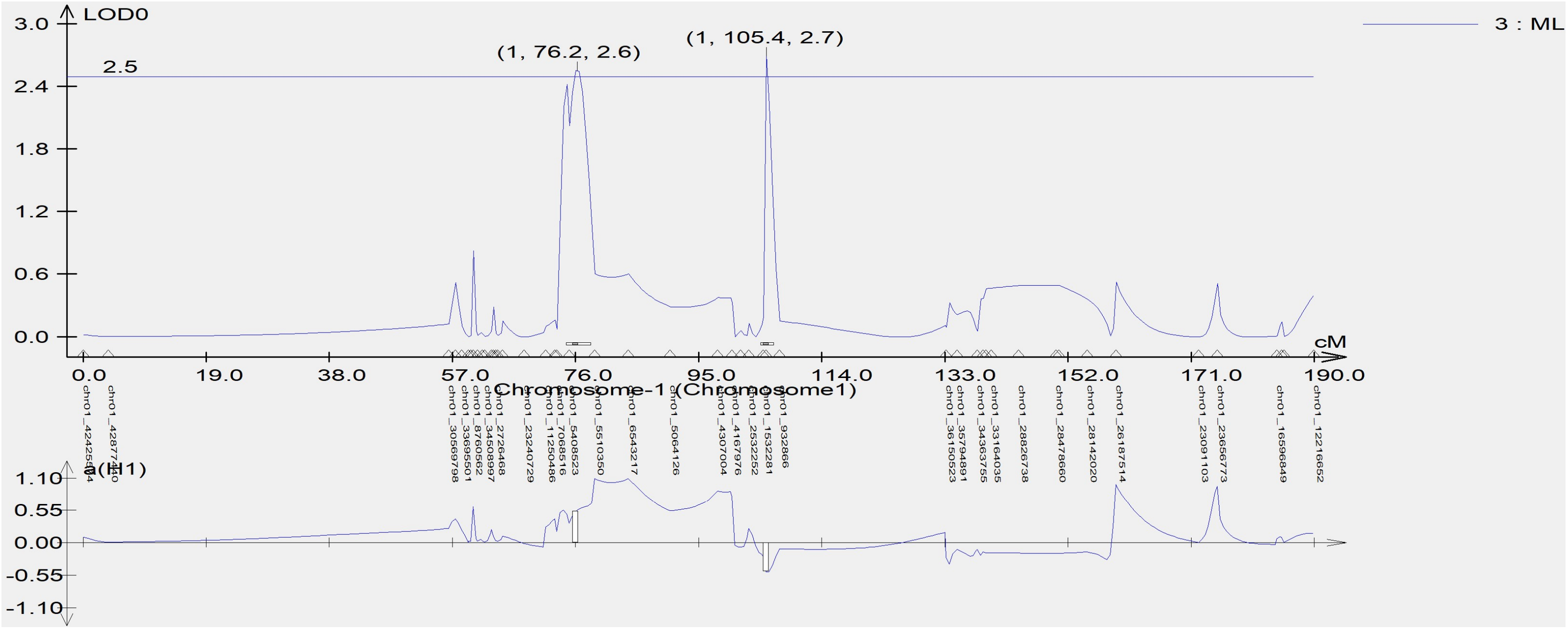
LOD0
3 : ML
(1, 76.2, 2.6)
(1, 105.4, 2.7)
2.5
cM
Chromosome-1 (Chromosome1)
a(H1)

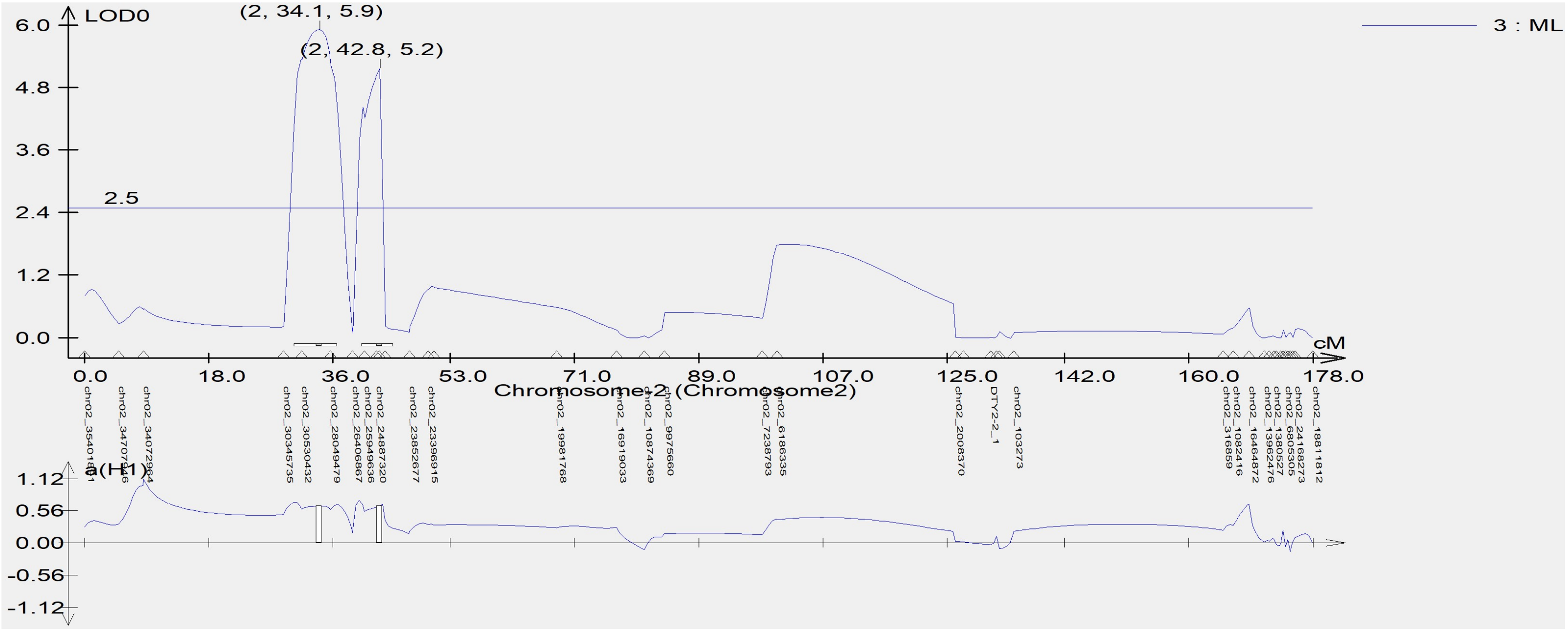
LOD0
3 : ML
(2, 34.1, 5.9)
(2, 42.8, 5.2)
2.5
cM
Chromosome-2 (Chromosome2)
a(H1)

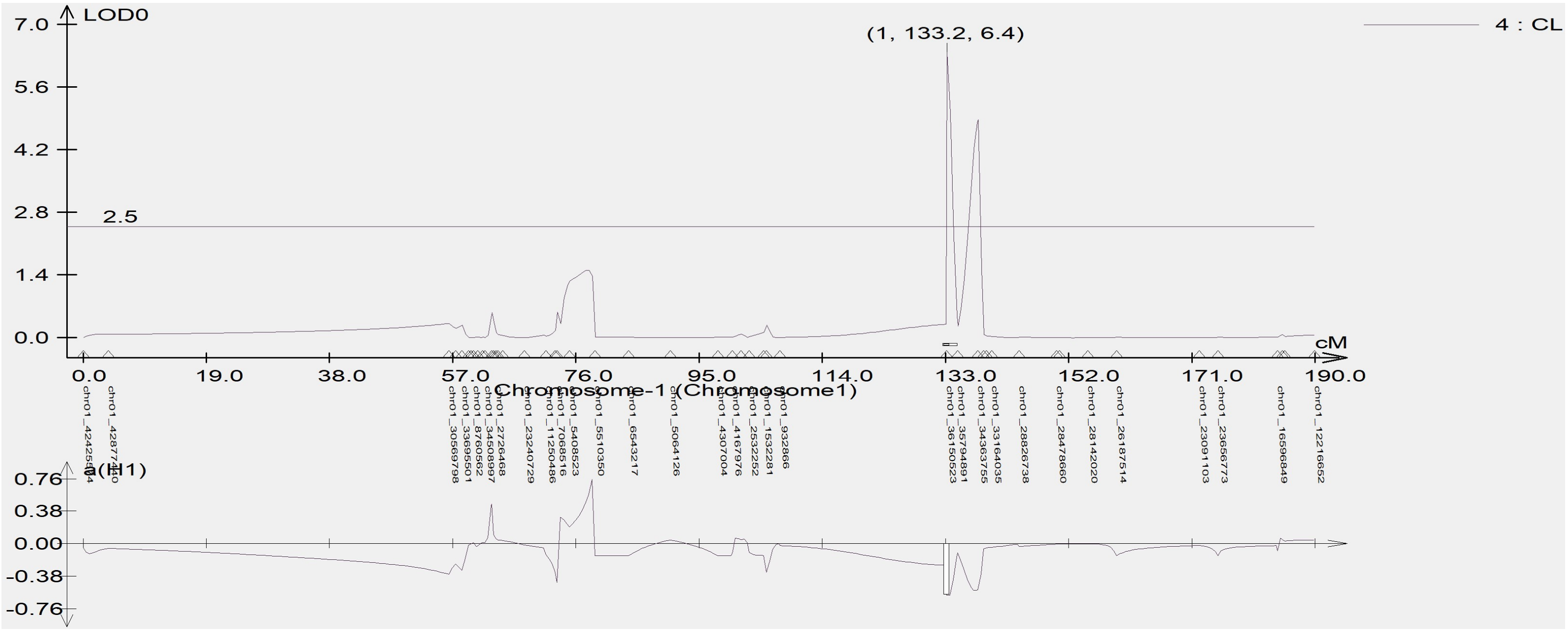
LOD0
4 : CL
(1, 133.2, 6.4)
2.5
cM
Chromosome-1 (Chromosome1)
a(H1)

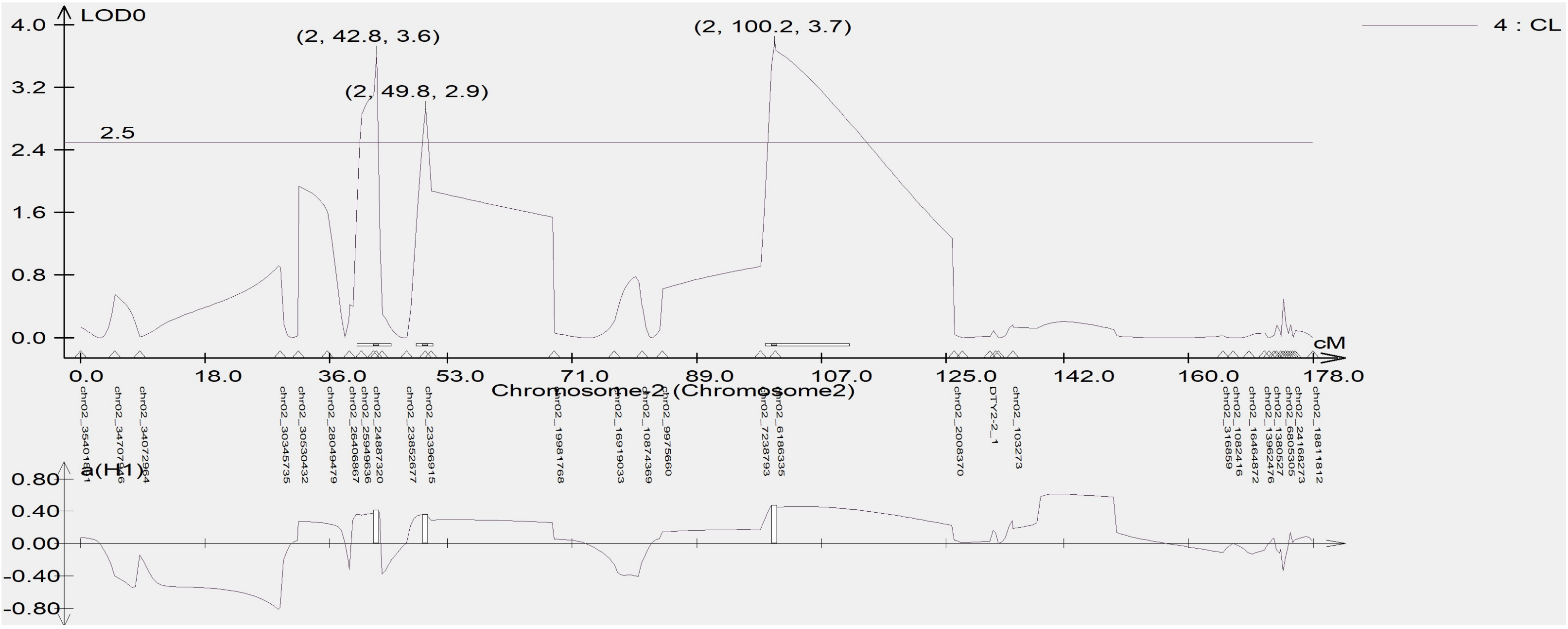
LOD0
4 : CL
(2, 42.8, 3.6)
(2, 49.8, 2.9)
(2, 100.2, 3.7)
2.5
cM
Chromosome-2 (Chromosome2)
a(H1)

QTL position using DS2023 phenotypic data

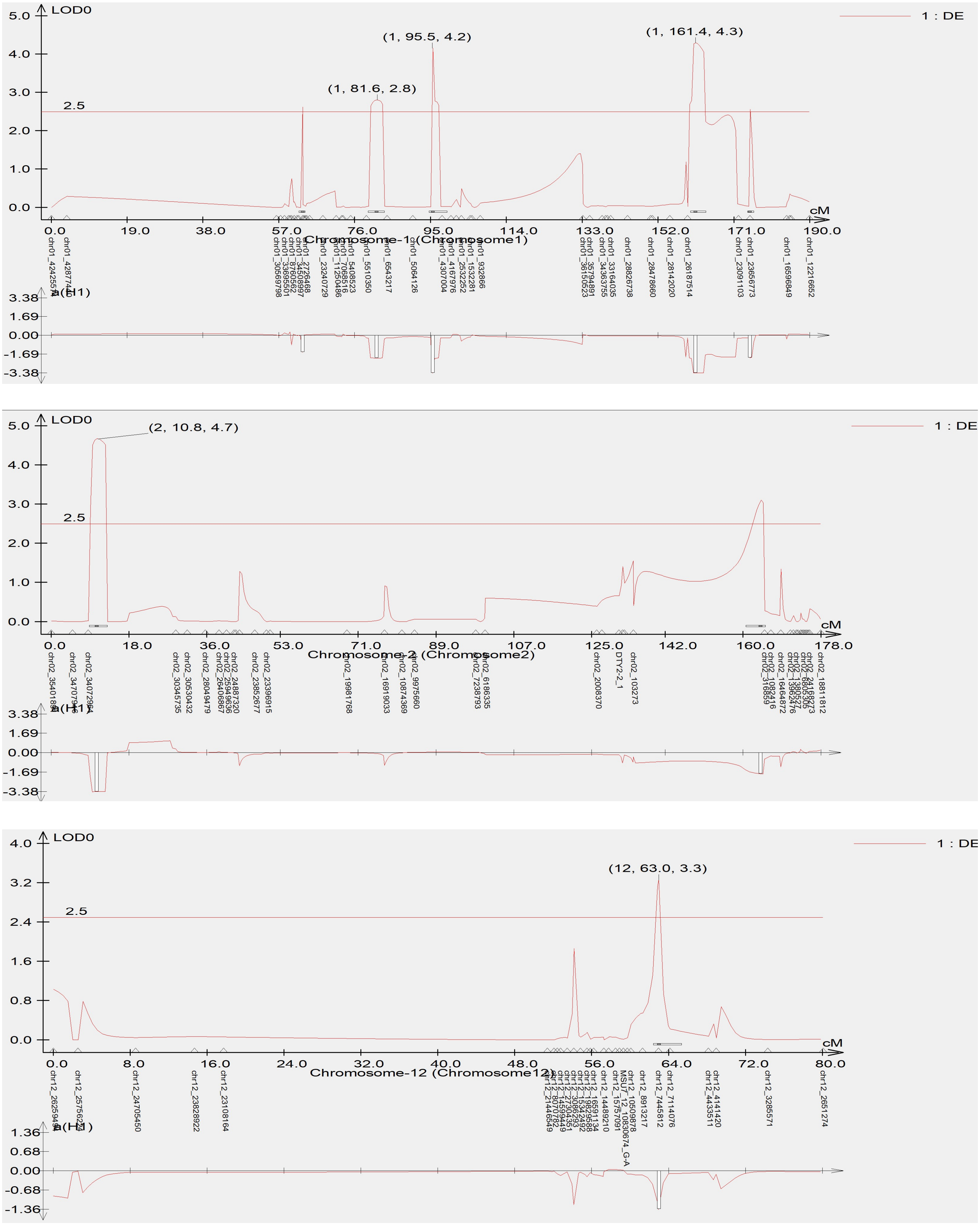

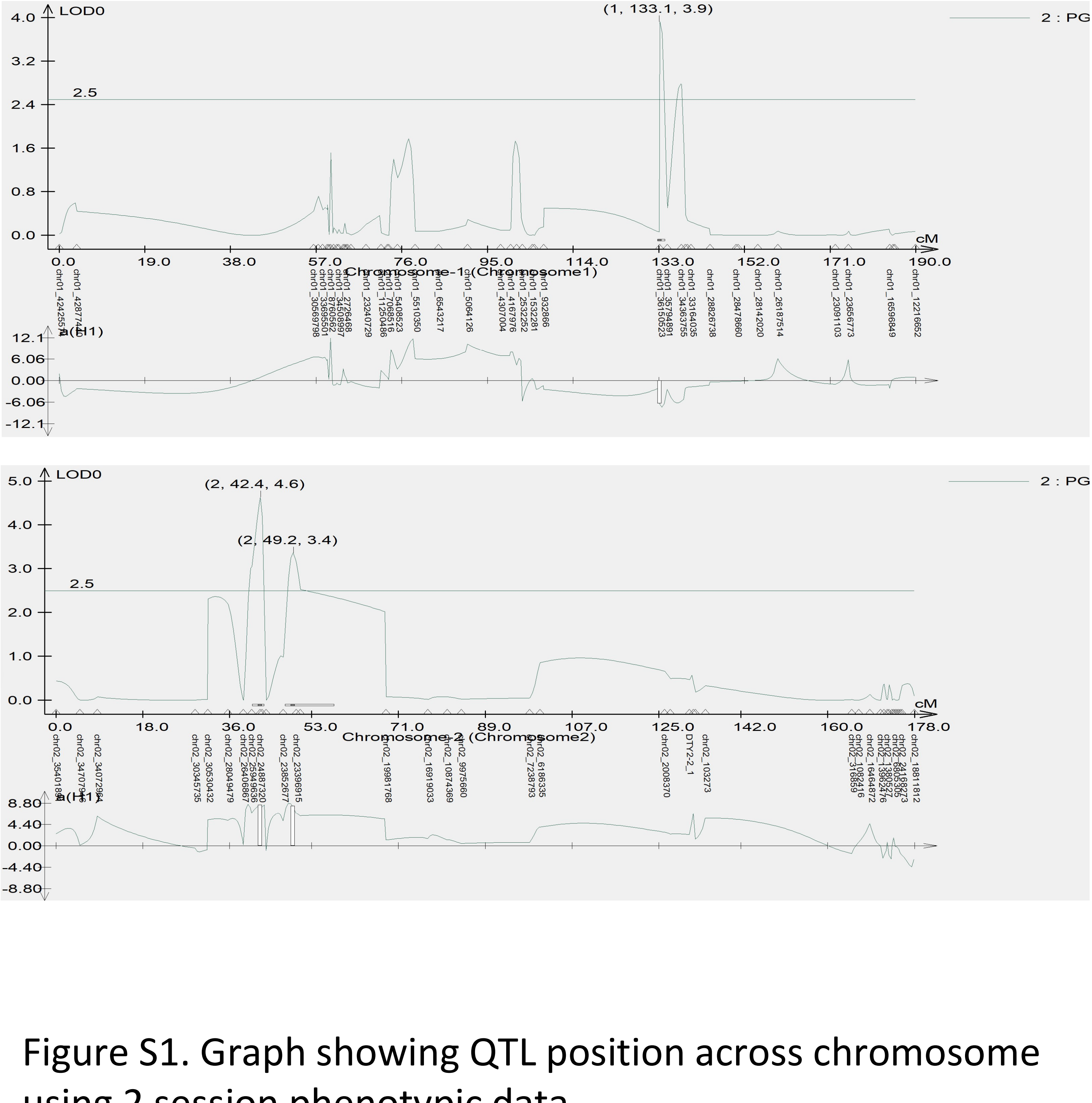

Figure S1. Graph showing QTL position across chromosome using 2 session phenotypic data.
