# Supplementary material for: Unveiling genetic basis of seedling emergence from deep soil depth under dry direct- seeded conditions in rice (Oryza sativa L.)
Source: Front Plant Sci. 2025 Jan 29;15:1512234. doi: 10.3389/fpls.2024.1512234 (PMC11814172; doi:10.3389/fpls.2024.1512234)
Supplement: Supplementary file 6 [file Image1.pdf]

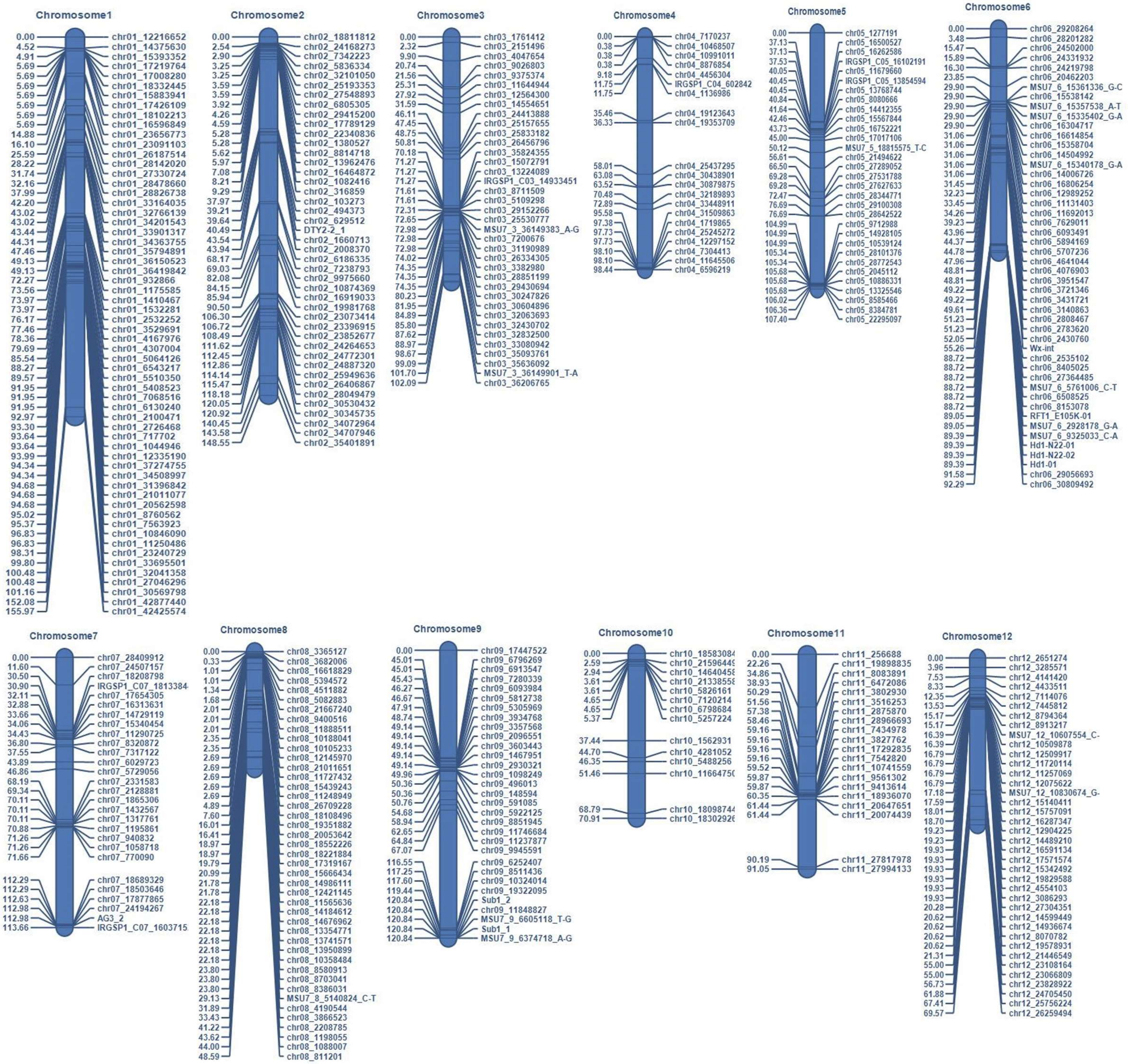

Figure S2. A genetic linkage map of 12 chromosomes was constructed using 414 SNP markers. This genetic map shows the distribution of markers on rice chromosomes. The map was built based on selected individuals of an F3:4 population of a cross between MTU1010 and AUS295. The chromosome number shown at the top of each linkage group. The names of the SNP markers are shown on the right, and cM position of marker showed at left side of chromosomes.
